# Development and validation of a checklist for cardiopulmonary bypass

**DOI:** 10.1051/ject/2024030

**Published:** 2025-03-07

**Authors:** Valdir Assis dos Reis Filho, Karina Aparecida Antonelli Novello, Mariana Leticia Matias

**Affiliations:** 1 Division of Cardiovascular Surgery, Instituto Dante Pazzanese de Cardiologia Av. Dr. Dante Pazzanese, 500 – Vila Mariana São Paulo – SP 04012-909 Brazil

**Keywords:** Cardiopulmonary bypass, Checklist, Patient safety

## Abstract

*Introduction*: Risk prevention protocols related to patient care have been developed to reduce the incidence of adverse events in health care services. Cardiopulmonary bypass (CPB) can trigger several complications, including physiological problems, electrical, mechanical, and human failures, or defective components. In order to promote patient safety and the identification of failures before they can cause damage, it is necessary to the use of a checklist for all surgeries. *Objective*: To prepare and validate a checklist for cardiopulmonary bypass. *Methodology*: A consensus validation methodology is applied in this study, in which the Delphi technique was structured for the instrument’s development. Five perfusion experts, with at least five years of experience, had an active participation in this research. First, a questionnaire was structured, based on a comprehensive review of the relevant literature. Then, two rounds of assessments were conducted, allowing for the collection of experts’ opinions. *Results*: In the first moment, a 42-item list was prepared and sent to the five experts for analysis. Based on participant’s responses, certain elements were accepted, excluded, and suggested. After that, a second 37-item list was assessed by the experts, resulting in all 37 items having an average assessment of ≥4 and a standard deviation ≤1.0 of acceptance. Based on these results, a 41-element checklist was developed, and these elements were considered crucial and relevant for the concerned analysis. *Conclusion*: The use of specific checklists for cardiopulmonary bypass comes into view as a highly proficient strategy, capable of promoting substantial improvements in the procedure safety and quality. The implementation and approval of this checklist should be considered.

## Introduction

Risk prevention protocols related to patient care have been developed to reduce the incidence of adverse events in health care services. These measures aim at promoting patient safety and the identification of failures before they can cause damage. Therefore, to develop efficient prevention actions, it is important to know the more critical processes that, as a consequence, are more prone to occur [1, 2].

Cardiopulmonary bypass (CPB) can trigger several complications that are mainly related to the interaction between blood and non-endothelial circuits of oxygenators, tubes, and reservoirs. The CPB effects on the body can result in endothelial edema, respiratory complications, neurological disorders, kidney injury, and post-surgery bleeding [3, 4]. CPB presents not only physiological changes, but also carries risks if done incorrectly, whether due to mistakes during the circuit assembly or defective components. Failures can be electrical or mechanical, encompassing a wide range of events, such as cracks, disconnections, air embolism, hemolysis, blood or gas flow obstruction, heat exchanger leakage or malfunctioning, and air blender malfunctioning [5, 6].

According to the guidelines proposed by the *Sociedade Brasileira de Cirurgia Cardiovascular* (SBCCV, Brazilian Society for Cardiovascular Surgery) and *Sociedade Brasileira de Circulação Extracorpórea* (SBCEC, Brazilian Society for Extracorporeal Circulation), the clinical perfusionist must use a checklist for all surgeries. These lists must be used as a checking guide, where each completed step must be checked and confirmed. Preferably, the checklist should be filled out by two individuals, one of them being the main perfusionist. However, if a second professional is not available at the time of checking, it is crucial to adopt a systematic-oriented routine of the list items to minimize the occurrence of adverse events [7, 8]. The American Society of Extracorporeal Technology (AmSECT), recommends that checklists shall be included as part of the patient’s permanent medical record [9].

Adequate preparation is essential to ensure patient safety during CPB, with patient safety considered inseparable from the quality of healthcare. European guidelines recommend the use of an institution-approved checklist before and during CPB, in the perioperative period (weaning from CPB, post-CPB, emergent reinstitution of CPB), and in procedures performed by clinical perfusionists [10]. AmSECT also recommends that the perfusionist should employ a checklist for additional perfusion services such as autotransfusion, intra-aortic balloon pump, and extracorporeal membrane oxygenation (ECMO) [9].

Human failure and poor maintenance of devices can contribute to the occurrence of accidents. The surgical team interaction, the early diagnosis, and the prompt intervention have shown to be crucial for a favorable outcome. Therefore, the clinical perfusionist plays a crucial role in cardiopulmonary bypass procedures, and their involvement is of utmost importance [6]. The perfusionist needs to adhere strictly to established procedures to ensure patient safety throughout the entire duration of CPB [11].

The assembly of the extracorporeal circuit and the inspection for potential failures in the perfusion equipment before its clinical use are important. The technological progress is evident, like the incorporation of alarms and fail-safe devices, but care continues to be vital for conducting CPB. However, the perfusionist’s attention to detail and adherence to pre-bypass checklists are still the foundation for a safe practice. Human error is the leading cause of accidents, even more than mechanical failure. CPB equipment test needs to be carried out to ensure roller pumps and heater/coolers are functioning well. As for the centrifugal pump console, it must be checked to observe potential electrical power failure; the most common method to do so is temporarily disconnecting the energy line from the console and checking if the backup battery alarm is fully operational. After the CPB circuit is assembled, the oxygenator and blood reservoir are placed in their proper support. The gas supply connection, the arterial flow pump tubes, and the roller lines should be safely connected; after that, the rollers must be properly calibrated. The two water lines of the heat exchanger are connected to the oxygenator, then water starts to flow to ensure there is no leakage [11, 12].

The checklist format is a process of continuous monitoring capable of developing efficient prevention actions, improving the cardiopulmonary bypass practice, and providing safer support to patients during a surgical procedure. The objective of the research was to develop and validate a checklist for cardiopulmonary bypass.

## Methodology

A validation methodology based on expert consensus was applied in this study, using the Delphi technique to develop and evaluate the instrument. The Delphi method, a structured communication process, involves multiple rounds of questionnaires, allowing experts to revise their responses based on feedback until a consensus is reached. In this study, two rounds were conducted to develop the checklist. The research was conducted from March to May 2023. The study was approved by the Research Ethics Committee of the Instituto Dante Pazzanese de Cardiologia (IDPC) – São Paulo, with the CAAE [Certificate of Presentation for Ethical Appreciation] number: 67490723.5.0000.5462.

Five clinical perfusionists with specialization recognized by the *Sociedade Brasileira de Circulação Extracorpórea* [Brazilian Society for Extracorporeal Circulation] and working in the area for at least 5 years have participated in the research. Each one of them was invited through an informative letter in which the study description and all stages were duly outlined. Also, the participants signed the Informed Consent Form (ICF) and were free to withdraw from the research at any moment.

An initial questionnaire was developed based on relevant contents selected according to the project’s purpose. A detailed review of the literature was made, focused on the pre-bypass care and aiming at finding the information required for a safe perfusion. Based on these findings, essential safety actions were identified in the cardiopulmonary bypass practice, which became the items to be checked for the instrument in making. This preliminary list had 42 items and, based on them, the instrument was sent to the subjects, and it was requested that they would accept or exclude the list stages during the first interaction, by marking YES or NO in the appropriate field. Also, the experts were encouraged to, if they wished to, include new tasks to the list, combine existing items, or provide further comments. This stage was the first Delphi questionnaire round, in which the responses were collected and analyzed, and the agreement and disagreement points were identified for the elements in the preliminary list. The score over 90% of acceptance would be kept on the list.

Based on the expert’s responses in the first round, a second list containing 37 items was prepared and sent once again to the subjects. A 1–5 scale was used to assess the importance and relevance of each item (1 = totally disagree and 5 = totally agree). The subjects were invited to review and reassess the scores, based on the results and feedback obtained in the previous round. A report was prepared with a summary of the results obtained in the first round.

The experts could attribute the score and had the opportunity to provide additional comments or rationale to the assessment. These comments were important to refine the understanding of results and provide valuable insights for the decision-making process. The comments and suggestions were considered in the refinement phase of the checklist.

According to the responses given by experts in the second round, the items that were not consistent with the statistical criteria of mean ≥ 4 and SD ≤ 1. 0 would be excluded from the checklist. The final version was based on these results.

## Results

The first round revealed a high agreement level between the five specialists when it comes to most of the items assessed in the questionnaire. Out of the 42 elements on the list, 38 had an approval score over 90%, marked as “YES”, showing strong consensus points among the specialists in the preliminary questionnaire. However, it is important to point out that 4 items (Custodiol Solution; Volts – 110/220; Level Sensor; Bubble Sensor) were scored under 90% or did not receive enough responses to determine a clear consensus. The percentage of approved items in a total of 42 was approximately 90.48% (38) and the percentage of non-approved items was approximately 9.52% (4). These results highlighted the need for a deeper analysis and additional discussions for the subsequent Delphi questionnaire rounds (Table 1).

**Table 1:** Preliminary list with 42 elements. The score over 90% of acceptance would be kept on the list.

Items version 1	Validity of the item
Patient	
01 Identification/Weight/Height	Valid
02 Surgical procedure	Valid
03 Calculations (Heparin/Flows)	Valid
04 Blood products checking	Valid
Equipment	
05 Power cables	Valid
06 Cooler	Rewritten/Repositioned
07 Heater	Rewritten/Repositioned
08 Volts – 110/220	Excluded
CPB Machine	
09 Initial test	Rewritten/Repositioned
10 Hand crank	Valid
11 Level sensor	Valid, not supported exclusion
12 Bubble sensor	Valid, not supported exclusion
13 Rollers	Rewritten/Repositioned
14 Water lines	Rewritten/Repositioned
15 Heat exchanger	Rewritten/Repositioned
Centrifugal pump	
16 Console	Valid
17 Flow sensor	Valid
18 Manual hand crank	Valid
19 Operational test	Valid
20 Driver	Rewritten/Repositioned
21 Pump battery	Rewritten/Repositioned
Gas supply	
22 O2 /Air regulation valve	Valid
23 Lines: O2 /Air (connected)	Valid
24 Blender – FIO2	Rewritten/Repositioned
25 Flowmeter	Rewritten/Repositioned
Materials	
26 Pharmacy kit	Valid
27 Oxygenator and components	Valid
28 Reynald clamps for tubes	Valid
Circuit preparation	
29 Safely connecting the tubes	Valid
30 Perfusate	Valid
31 Calibration of the arterial roller and aspirators	Valid
32 Air Removal from the system	Valid
33 No Leakage in the circuit	Valid
Cardioplegia	
34 Test in the cardioplegia module	Valid
35 Circuit assembly/Checking	Valid
36 Cardioplegia solution	Valid
37 Custodiol solution	Excluded
Vacuum system	
38 Vacuum regulator	Valid
39 Relief valve	Valid
Before starting CPB	
40 Heparin dosage	Valid
41 Anticoagulation/ACT	Valid
42 Arterial line test	Valid

CPB – cardiopulmonary bypass; ACT – activated coagulation time.

Therefore, 2 items (Custodiol Solution; Volts – 110/ 220) were excluded for not complying with the 90% of acceptance condition, and 4 (Pathology; Equipment Clean; Machine Battery; Ice) were included as a suggestion by the specialists. Ten elements were rewritten or repositioned (Heater; Cooler; Initial Test; Rollers; Water Lines; Heat Exchanger; Driver; Pump Battery; Blender – FIO2, Flowmeter), resulting in a more comprehensive data, and 2 (Level Sensor; Bubble Sensor) items from the previous round under 90% of approval were kept in the second list, considering that, even though they are not included in the reality of the professionals, they may represent a highly potential perspective in the future. After the analysis of the second round results, all 37 items reached a mean ≥ 4 and SD ≤ 1. 0 (Table 2). Three items were additionally suggested for inclusion in the final checklist.

**Table 2:** Final list with 37 elements that were assessed as mean ≥ 4 and SD ≤ 1.02.

Items version 2	Mean	SD
Patient		
01 Identification/Weight/Height	4.4	0.54
02 Pathology	4.4	0.54
03 Surgical procedure	4.4	0.89
04 Calculations (heparin/flows)	4.8	0.44
05 Blood products checking	4.8	0.44
Equipment		
06 Equipment clean	4.6	0.54
07 Power cables	4.6	0.89
08 Heater-cooler	4.8	0.44
CPB machine		
09 Initial test (Rollers/Water lines/Heat exchanger)	4.8	0.44
10 Hand crank	4.8	0.44
11 Level sensor	4.0	0.63
12 Bubble sensor	4.2	0.44
13 Battery	4.6	0.89
Centrifugal pump		
14 Console/Driver/Battery	4.4	0.89
15 Flow sensor	4.8	0.44
16 Manual hand crank	4.8	0.44
17 Operational test	4.4	0.54
Gas supply		
18 O2/air regulation valve	4.6	0.54
19 Lines: O2/Air (Connected)	4.6	0.54
20 Blender/Flowmeter/FIO2	4.6	0.54
Materials		
21 Pharmacy kit	4.6	0.54
22 Oxygenator and components	4.4	0.89
23 Reynald clamps for tubes	4.4	0.89
24 Ice	4.6	0.89
Circuit preparation		
25 Safely connecting the tubes	4.8	0.44
26 Perfusate	5.0	0.0
27 Calibration of the arterial roller and aspirators	4.4	0.54
28 Air removal from the system	4.6	0.54
29 No leakage in the circuit	4.4	0.54
Cardioplegia		
30 Test in the cardioplegia module	4.2	0.83
31 Circuit assembly/checking	4.4	0.54
32 Cardioplegia solution	4.2	0.97
Vacuum system		
33 Vacuum regulator	4.6	0.89
34 Relief valve	5.0	0.0
Before Starting CPB		
35 Heparin dosage	4.4	0.54
36 Anticoagulation/ACT	4.6	0.54
37 Arterial line test	5.0	0.0

*CPB – cardiopulmonary bypass; ACT – activated coagulation time.

**Figure 1:**
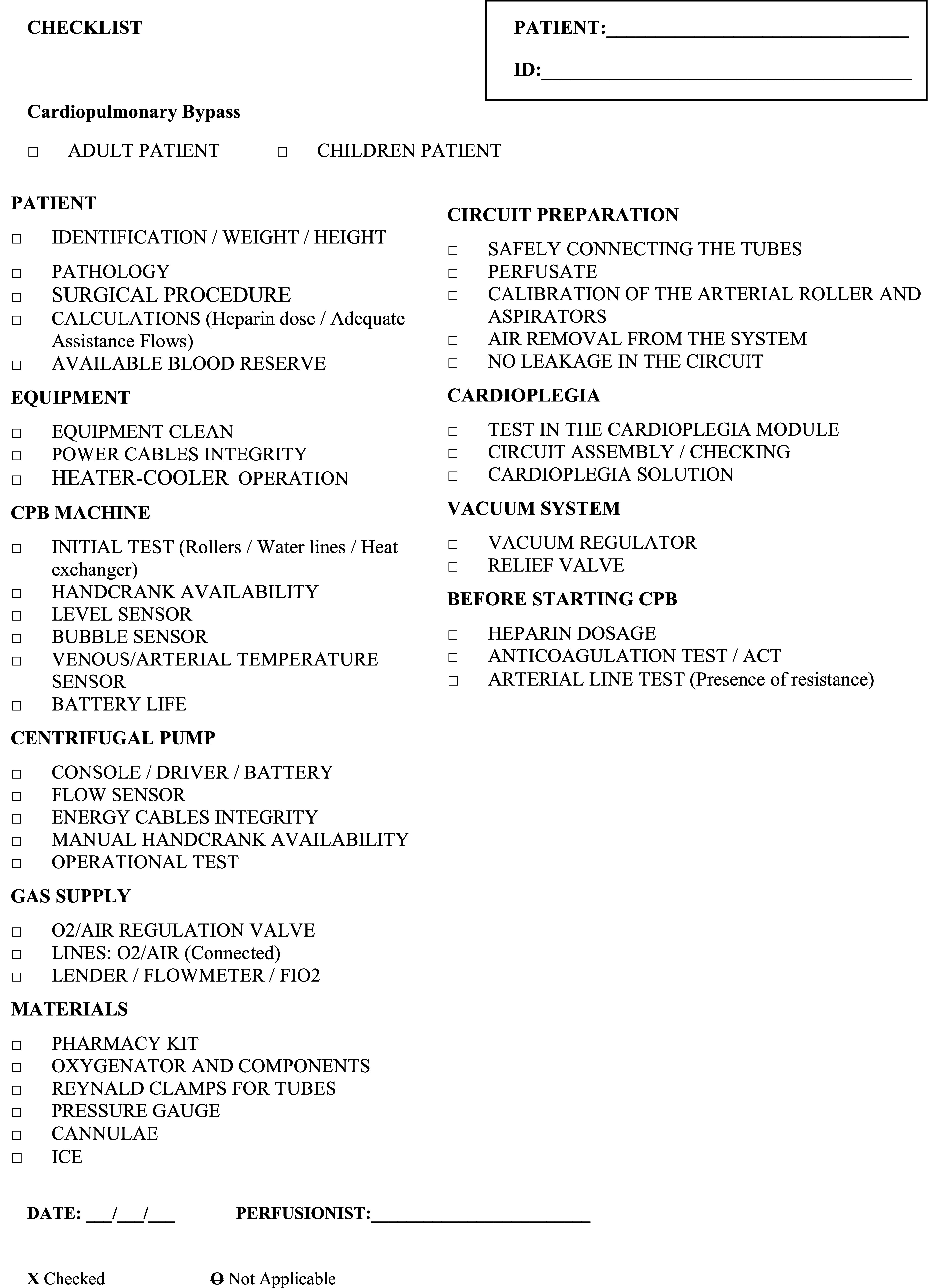
The final version of the cardiopulmonary bypass checklist with 10 topics and 41 carefully selected checking items.

Based on the results obtained in the last round, a cardiopulmonary bypass checklist has been developed. This document covers all items and checks required, from identification of components to checking devices and operational tests. The items were divided into 10 topics and 3 elements were included in the last round after suggestion by the specialists: venous/ arterial temperature sensor, pressure gauge, and cannulae) (Figure 1).

## Discussion

The list we have developed encapsulates all the pre-bypass moments, from preparing the equipment to the initial procedure, showing aspects such as functional device tests, identification of circuit components, preparation of equipment, and observing potential CPB-related failures. The purpose of the checklist is to ensure the proper execution of essential steps and necessary verifications for the efficient functioning of the system. The guidelines published by the Brazilian Society for Cardiovascular Surgery and Brazilian Society for Extracorporeal Circulation [8] as well as the perfusion checklist developed by the American Society of Extracorporeal Technology [9] propose that the perfusionist use this checklist in all CPB procedures, and to include it in the patient’s medical chart. SBCEC and SBCCV recommend the use of a checklist throughout the perioperative period, which would involve not only the pre-bypass period but also the circulatory assistance period and post-bypass; a document in which the perfusionist highlights all pieces of information related to this period has been already established and used, which is called the “perfusion sheet”. This article is specifically focused on preparing a checklist for the period prior to the extracorporeal procedure starting, and we consider that we have attained this goal.

Standardization is one of the main benefits of the CPB checklist in cardiovascular surgery. CPB involves a set of devices and interconnected stages, and detail is crucial for the system’s operation. The checklist provides a systematic and consistent guide, which aims at limiting the possibilities of errors that are often caused by not identifying issues earlier or even by the negligence of the professionals. Nicoletti [11] points out important elements to develop a checklist, with nine topics suggested being crucial: (1) Patient identification data, (2) equipment, (3) materials, (4) surgery planning, (5) circuit preparation, (6) prime, (7) anticoagulation, (8) macro monitoring and patient micro-hemodynamics (9) temperature. The items suggested by Nicoletti [11] contain elements focused on the period pre- and intra-surgical. AmSECT [12] recommends using the perfusion checklist developed by them with the topics: of weaning/termination (VAVD off) and post bypass (announce bypass terminated), or a reasonable equivalent. Our study focuses on the pre-CPB phase and highlights ten essential topics designed to enhance patient safety through early detection of adverse events. By carefully checking each item on the checklist, we enable the prompt identification of any failures before the procedure begins. This proactive approach allows for swift and effective interventions, ensuring continuity in the process.

The technical complexity and interdependence of devices require a systemic approach, in which every step is consistently and precisely carried out. It is essential to integrate this practice into the professional’s routine, making it a habit process. The standardization after checking all elements in the cardiopulmonary bypass technique may reduce the variability between professionals. Future studies must analyze the ease of use, execution time, and team engagement with the developed checklist.

While checklists are crucial for ensuring safety and consistency in clinical perfusion practice, excessively long or redundant checklists can lead to disengagement and errors from routine memorization. Drawing parallels to the aviation industry, where checklist fatigue has been well-documented, we will suggest focusing on critical parameters directly related to patient safety and eliminating redundant steps. This selective approach ensures that the checklist remains an effective tool for error prevention without overwhelming the user.

A limitation of the study was the number of participating judges. Five perfusionists were included in the research from the same center/service, however, it would be important to expand the criteria used to compose the sample of experts. The involvement of professionals from more than one health service and the participation of some professionals who work in the surgical environment, such as cardiac surgeons and anesthesiologists, would also be interesting.

## Conclusion

The use of specific cardiopulmonary bypass checklists comes into view as a highly proficient strategy, capable of promoting substantial improvements in the procedure safety and quality, being vital to ensure excellence in care for patients undergoing cardiovascular surgeries. The implementation and approval of this checklist should be considered as a practice recommended in cardiovascular surgery-aimed institutions and services, aiming at providing safer and more successful results.

## Data Availability

The research data are available on request from the authors.
