# Corrigendum to: Development and validation of a checklist for cardiopulmonary bypass

**DOI:** 10.1051/ject/2025037

**Published:** 2025-09-15

**Authors:** Valdir Assis dos Reis Filho, Karina Aparecida Antonelli Novello, Mariana Letícia Matias

**Affiliations:** 1 Division of Cardiovascular Surgery, Instituto Dante Pazzanese de Cardiologia, Av. Dr. Dante Pazzanese 500 – Vila Mariana São Paulo – SP 04012-909 Brazil

After publication, the corresponding author identified two minor typographical errors that were not corrected prior to finalization:

**1. In Table 2**, the text currently reads:

*“Final list with 37 elements that were assessed as mean ≥ 4 and SD ≤ 1. 02.”*

The correct form should be:

*“Final list with 37 elements that were assessed as mean ≥ 4 and SD ≤ 1.0.”*

**2. In Figure 1**, the text currently reads:

*“LENDER / FLOWMETER / FIO2”*

The correct form should be:

*“BLENDER / FLOWMETER / FIO2”*

